# ANMAF: an automated neuronal morphology analysis framework using convolutional neural networks

**DOI:** 10.1038/s41598-021-87471-w

**Published:** 2021-04-14

**Authors:** Ling Tong, Rachel Langton, Joseph Glykys, Stephen Baek

**Affiliations:** 1 Department of Business Analytics, University of Iowa, Iowa City, 52242 Iowa United States; 2 Departments of Pediatrics and Neurology, Iowa Neuroscience Institute, University of Iowa, Iowa City, 52242 Iowa United States; 3 Department of Industrial and Systems Engineering, University of Iowa, Iowa City, 52242 Iowa United States

**Keywords:** Image processing, Computational neuroscience

## Abstract

Measurement of neuronal size is challenging due to their complex histology. Current practice includes manual or pseudo-manual measurement of somatic areas, which is labor-intensive and prone to human biases and intra-/inter-observer variances. We developed a novel high-throughput neuronal morphology analysis framework (ANMAF), using convolutional neural networks (CNN) to automatically contour the somatic area of fluorescent neurons in acute brain slices. Our results demonstrate considerable agreements between human annotators and ANMAF on detection, segmentation, and the area of somatic regions in neurons expressing a genetically encoded fluorophore. However, in contrast to humans, who exhibited significant variability in repeated measurements, ANMAF produced consistent neuronal contours. ANMAF was generalizable across different imaging protocols and trainable even with a small number of humanly labeled neurons. Our framework can facilitate more rigorous and quantitative studies of neuronal morphology by enabling the segmentation of many fluorescent neurons in thick brain slices in a standardized manner.

## Introduction

Neurons have complex histology with multiple branches, rendering considerable challenges in computing somatic and dendritic sizes during normal and pathological conditions, including seizures, hypoxia, extracellular osmolarity fluctuations, etc^1–3^. Most studies rely on manual or pseudo-manual methods to measure somatic areas^1,4–6^, which have considerable drawbacks: they are labor-intensive, prone to human bias, and susceptible to intra- and inter-observer variances. Variances are particularly prominent when establishing where the soma ends, and the dendritic or axonal processes start. These shortcomings can significantly and negatively impact statistical analyses of neuronal size fluctuations during physiological and pathological conditions.

Recent studies have developed fully automated methods to detect or segment cells in 2D images of fixed tissue slices, neuronal cultures, or brain slices^7–12^. These methods span from traditional image processing approaches relying on hand-crafted features to modern CNN-based procedures that can automatically learn practical features and parameters from data. Most recently, Mask R-CNN^13^, a popular method for object detection and instance segmentation, has demonstrated its performance in multiple challenges, including automatic detection and counting of retina cell nuclei^12^, segmentation of immune cells in human lupus nephritis biopsies^14^, and neuroanatomical image segmentation^11^.

However, despite previous studies in cell detection and segmentation tasks, the efficacy, reliability, and generalizability of CNNs in neuronal morphology analysis remain understudied. For one, statistical metrics such as F1 score and Sørensen-Dice coefficient that are commonly used in machine learning or computer vision literature have not been interpreted in the context of neuronal morphology analysis. Hence, it remains unresolved, for most neuroscientists, the implication of performance evaluations of CNNs, i.e., how much they agree with human annotators, how reliable they are, etc. Moreover, the practicality of CNNs is also in doubt, as neuronal images with complete and exhaustive annotations (e.g., labels and contours) are available only in a small quantity, or not at all. Notably, it is far from feasible to achieve complete and exhaustive human annotations of every neuron in each of the acquired experimental images due to various practical reasons. These reasons include overlapping neurons over different depth planes, the difference in fluorescence intensity over the thickness of a brain slice, low contrast between cell bodies and the background, etc. CNN models are data-hungry, and incomplete annotations with unlabeled somatic regions confuse a CNN model during training and deteriorate its performance.

Here, we present a novel high-throughput Automated Neuronal Morphology Analysis Framework, named ANMAF, built around Mask R-CNN to automatically contour fluorescent neuronal somatic areas in acute brain slices (abbreviated as ‘*cell images’*). Using two-photon images of brain slices with neurons expressing the genetically encoded fluorophore Clomeleon (YFP channel^15,16^), as an input, ANMAF detects and exports somatic contours to an ImageJ (National Institutes of Health, United States) format. Due to a novel data generation pipeline, training of ANMAF only requires a small number of human-labeled neurons. ANMAF exhibits high reliability in identifying fluorescent-labeled neurons without human bias and it is generalizable to different imaging devices and protocols.

## Results

### The ANMAF framework

**Figure 1:**
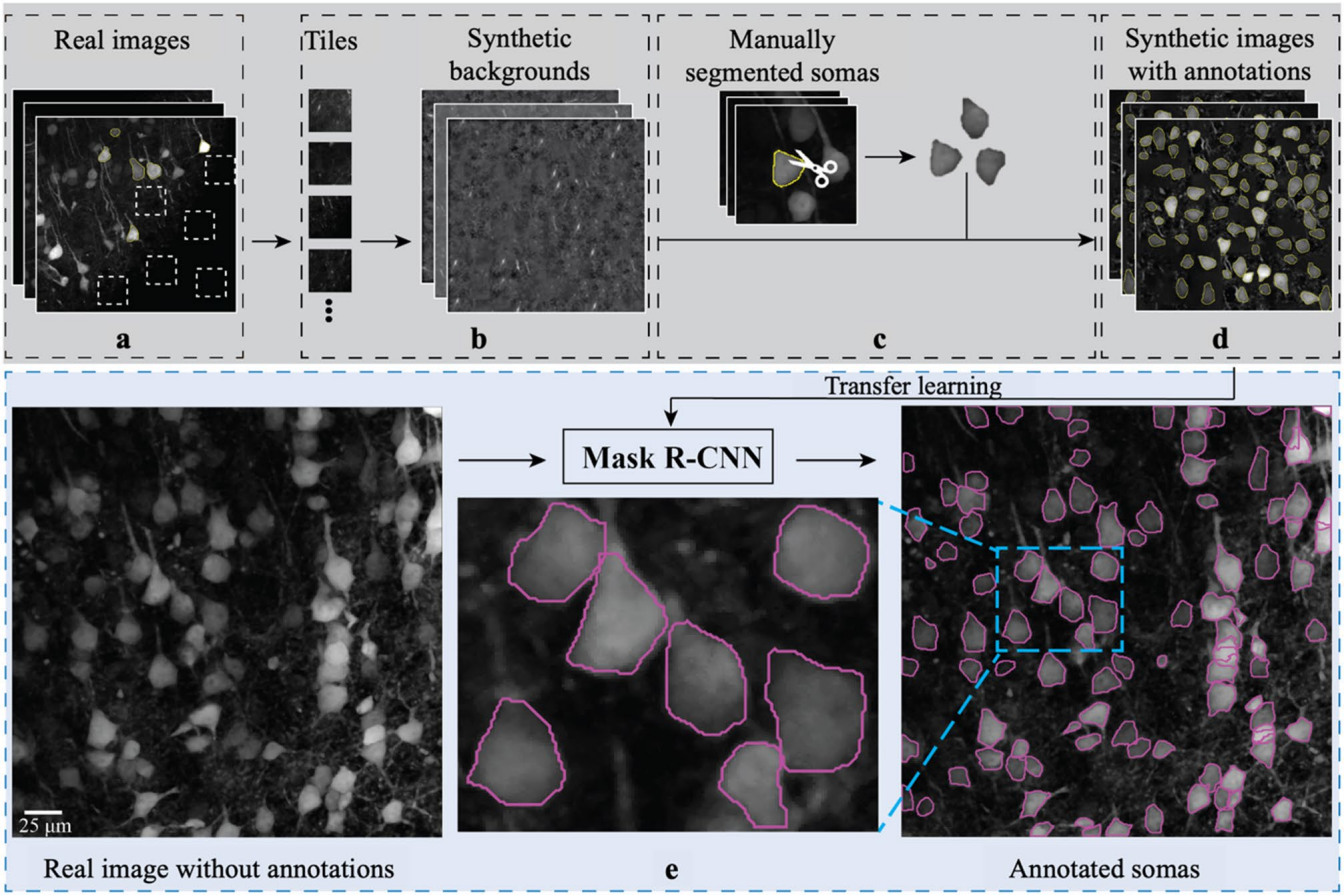
ANMAF development. (**a**) A library of background tiles was constructed from the background of multiple two-photon images. (**b**) The background tiles were shuffled and arbitrarily placed to produce a collection of random synthetic backgrounds. (**c,d**) Somatic areas from the original human detected neurons were cut, pasted, and blended into randomly selected backgrounds to generate multiple synthetic images. (**e**) The Mask R-CNN model was initiated with parameters transferred from a pre-trained model and fine-tuned with synthetic images from the previous steps. Once trained, the ANMAF generated somatic contours from a given neuronal input image.

As in other deep learning methods, Mask R-CNN demands many manually annotated data samples for training. Existing neuronal morphology data sets from prior studies are unsuitable for training Mask R-CNN. They have an insufficient number of images, and incomplete labeling of neurons as human annotators only labeled a small subset of neurons in each image. To address this issue, we implemented within ANMAF an automated algorithm to generate synthetic cell images from partially annotated two-photon microscopy images of the YFP channel of Clomeleon expressing neurons from acute brain slices. We first sampled empty regions in two-photon microscopy images to create background tiles (Fig. 1a). Next, we constructed a library of synthetic backgrounds by placing the background tiles at random locations (Fig. 1b). The edges of a tile were blended into its neighboring tiles to avoid unnatural contrasts. Meanwhile, somatic areas were cropped along their contours from the original microscopic images, wherever manual annotations were available (Fig. 1c). These cropped areas were then randomly transformed, pasted, and blended in at arbitrary locations of randomly selected synthetic backgrounds (Fig. 1d). Afterward, ANMAF was trained on these synthetically generated images. Model parameters were initialized by transferring values from a pre-trained Mask R-CNN on a generic object detection data set in computer vision, called the Common Objects in Context (COCO)^17^. With these generic parameters as a starting point, we employed the optimizer gradient descent with momentum^18^ to search for a specific parameter configuration that minimizes the discrepancy between human-labeled contours and the automatically detected contours in the synthetic cell images. Once trained, ANMAF was applied on real cell images to obtain neuronal somatic region annotations (Fig. 1e).

### Validation

**Figure 2:**
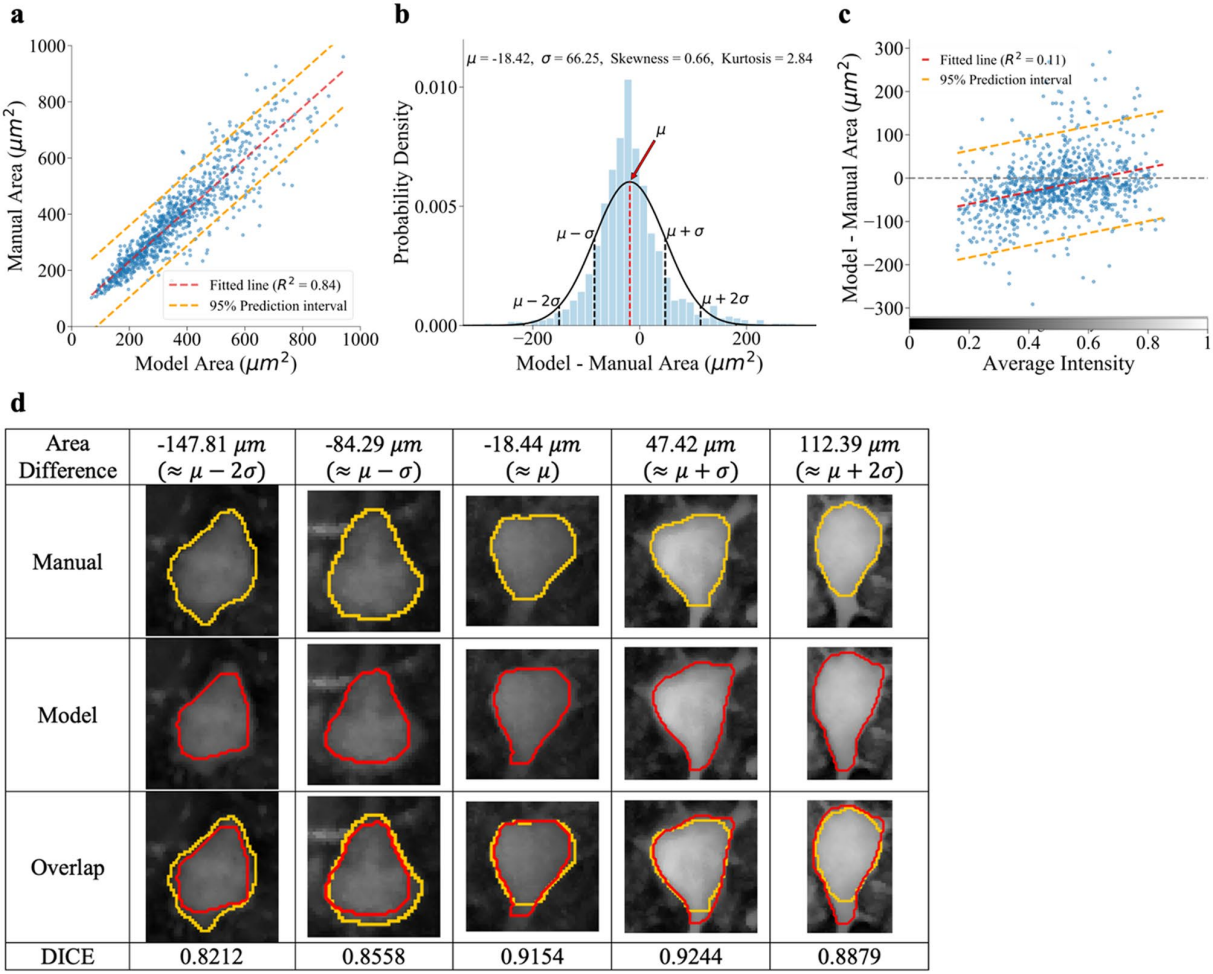
ANMAF performed similarly to manual annotators. (**a**) Correlation between manual area and model area. (**b**) Probability density of the differences between model and manual area. The black curve is the Gaussian fit. Blue bars represent the distribution of the actual data. (**c**) Neurons’ average intensity versus area difference with the standard least square regression and 95% prediction interval. (**d**) Examples of manual and model area differences.

After training, we tested the performance of ANMAF by comparing its detection results with those of human annotators on real cell images. We evaluated the agreement between ANMAF and human annotators in terms of yield rate (percentage of manually annotated neuronal somatic regions detected by ANMAF), conformity of contours, and the agreement in area measurements. To determine the yield rate, we searched for the most overlapped somatic region detected by ANMAF for each manually annotated somatic region using the Sørensen-Dice coefficient (DICE)^19^. A match occurred if the highest DICE was greater than 0.5. Out of 965 manually identified neurons, ANMAF detected 926 neurons (96% yield rate). Among these 926 neurons, the average conformity between human and model-generated annotations was 0.88, measured using DICE. We also compared the area measurements between the human annotators and ANMAF by applying the shoelace algorithm^20^ on contours. There was a positive correlation between manual measurements and model-generated measurements (Pearson *r* = 0.917, CI [0.906, 0.927], ${R}^{2}$ = 0.84, *p* < 0.0001, *n* = 926; Fig. 2a). The average difference between model and manual areas was − 18.42 ± 66.25 μm^2^ (mean ± SD, CI [− 22.7, − 14.2]; *p* < 0.0001, paired *t*-test; Fig. 2b), implying that the areas measured by ANMAF were slightly smaller than those measured by human annotators. One primary source of difference originated from the human annotators’ variability when the soma ends and when the dendritic/axonal processes start (Fig. 2d). Also, there was a weak positive correlation for ANMAF to underestimate the somatic area in dimmer neurons and overestimate the area in brighter neurons (Pearson *r* = 0.326, CI [0.268, 0.383], *R*^2^ = 0.11, *p* < 0.0001; Fig. 2c,d). This tendency can be understood since human annotators were allowed to manipulate image intensity and contrast proactively during contouring and to examine adjacent slices to obtain contextual information. In contrast, ANMAF had no flexible control over the image properties nor access to adjacent slices when contouring somatic regions on a single slice. In the course of the study, we also assessed the performance variation of ANMAF. We trained ten models on synthetic images generated using different random seeds. Weights were re-initialized at the beginning of the training process for each model and the training images were shuffled after each epoch. Overall, these ten models had a stable performance across all real images. The average yield rate was 96.06% ± 0.37% (SD). The conformity of manual contours and model-generated contours was on average 0.8882 ± 0.0056 (SD), and the correlation between manual and model-generated area measurements was on average 0.9098 ± 0.0041 (SD).

**Figure 3:**
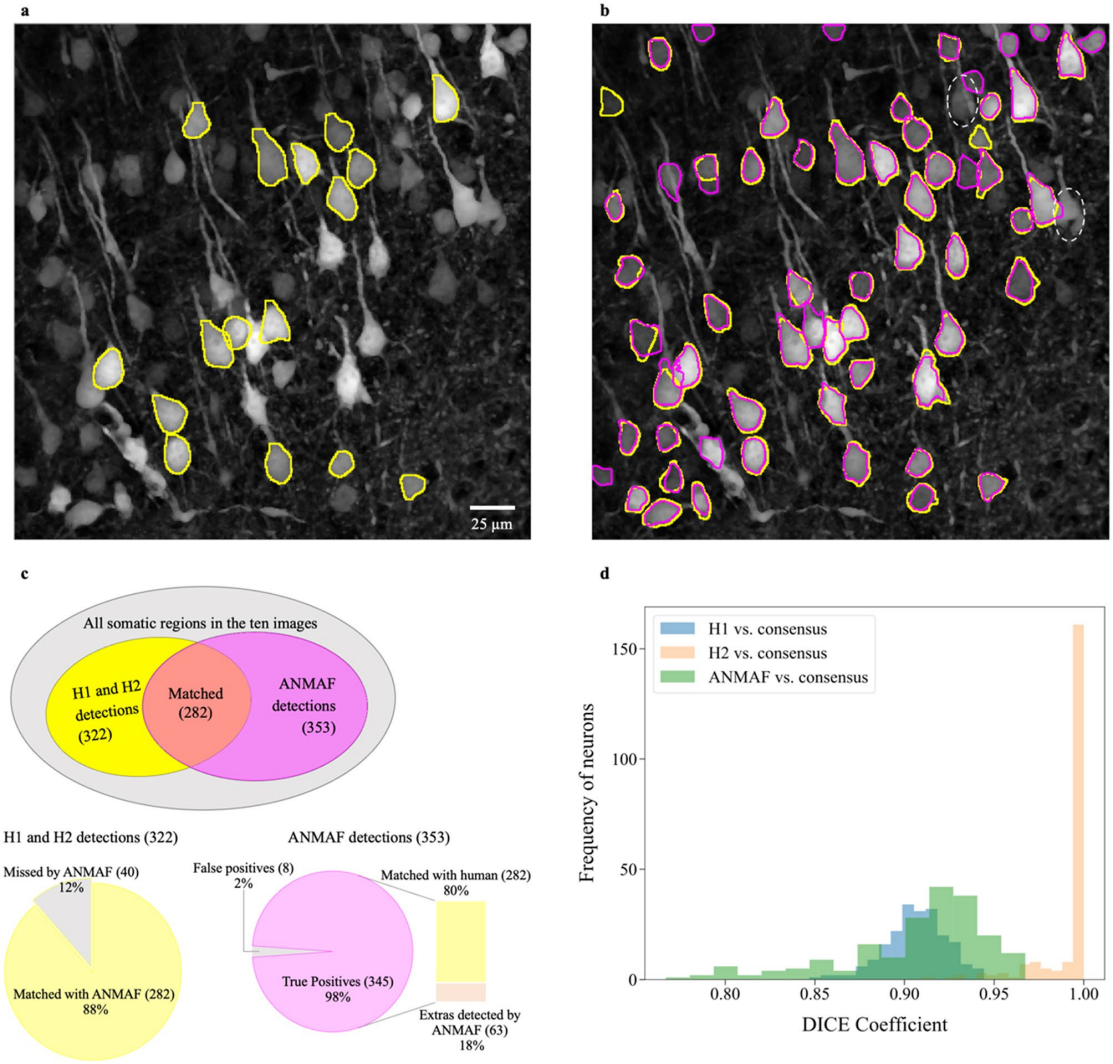
ANMAF exhibited high precision. (**a**) A selected real image with original annotations. Human annotators initially contoured only a small subset of visually prominent somatic regions. (**b**) The same image was relabeled by two human annotators to contour as many somatic regions as possible to compare with ANMAF. Yellow, human-labeled contours; Magenta, ANMAF generated segmentations. White dashed ellipses indicate two somatic regions that both the human annotators and ANMAF could not segment. (**c**) Human H1 and H2 together identified 322 somatic areas, and ANMAF detected 353 ROIs from the ten images. There were 283 regions in common. Note that neither the human annotators nor ANMAF identified every single somatic region. ANMAF detected 88% of regions labeled by the human annotators. Among the 353 somatic ROIs detected by ANMAF, 98% of them were somatic regions. (**d**) DICE coefficient histogram of H1, H2, and ANMAF against the human consensus using the STAPLE algorithm. ANMAF performance was between H1 and H2.

Across all real images, human annotators only labeled a subset of visually prominent somatic regions (965) due to the labor-intensive process of manual labeling (Fig. 3a). However, ANMAF detected 3808 regions of interest (ROIs). To further determine the performance of ANMAF, two human annotators (H1 and H2) had the task of relabeling as many somatic areas as possible in a set of ten randomly selected real images. Obtaining a comprehensive ‘gold standard’ from multiple human annotations is costly, time-consuming, and challenging. In neurons that overlap or change their contrast or fluorescent expression, it is near impossible. Despite the instructions to find all somatic regions as thoroughly as possible, the two annotators could not find them all, nor they had a perfect agreement. Even with flexible control over image intensity and contrast during contouring, the human annotators could not label some overlapping neurons, edge neurons, and dimmed neurons as the neurons were either ambiguous to the human labelers, they had an incomplete cell body boundary, or the human annotators were not confident in marking the correct edge (Fig. 3b). Due to higher labeling confidence, H1 labeled 315 somatic regions, but H2 only annotated 213 areas. There were 206 neurons in common, marked by both H1 and H2.

The two human annotators collectively contoured 322 unique somatic regions, and ANMAF segmented 353 somatic ROIs. Although ANMAF detected a few more ROIs, it did not find all possible areas (Fig. 3b,c). Out of the 322 manually labeled regions, 282 were also detected by ANMAF (Fig. 3c). We manually checked the rest of the 71 ROIs segmented by ANMAF and found that 63 were cell bodies with unclear visual boundaries (e.g., dimmed neurons, overlapping neurons, or edge neurons). Overall, 345 of the 353 model-detected ROIs were indeed cell bodies (true positives, precision 0.98; Fig. 3c). Among all areas labeled, there were 199 regions segmented by all three annotators (H1, H2, and ANMAF). We computed the consensus of human annotators for the 199 areas using the STAPLE algorithm ^21^. We then compared ANMAF, H1, and H2 against the human agreement using the DICE coefficient (Fig. 3d). H2 tends to be more conservative than H1 as most of the regions contoured by H2 were contained in the areas annotated by H1. Therefore, the consensus produced by the STAPLE algorithm is very similar to H2’s annotations. Compared with the consensus, ANMAF had its performance centered between the two human labelers H1 and H2 (Fig. 3d). The absolute average difference between H1 and H2 measured area was 55.54 $\pm$ 27.29 μm^2^, while the difference between ANMAF and the consensus area was 18.81 $\pm$ 48.24 μm^2^. Thus, on average ANMAF had a smaller area difference against a consensus measurement compared to the area discrepancy between human annotators.

### Variability and reproducibility during repeated measurements

**Figure 4:**
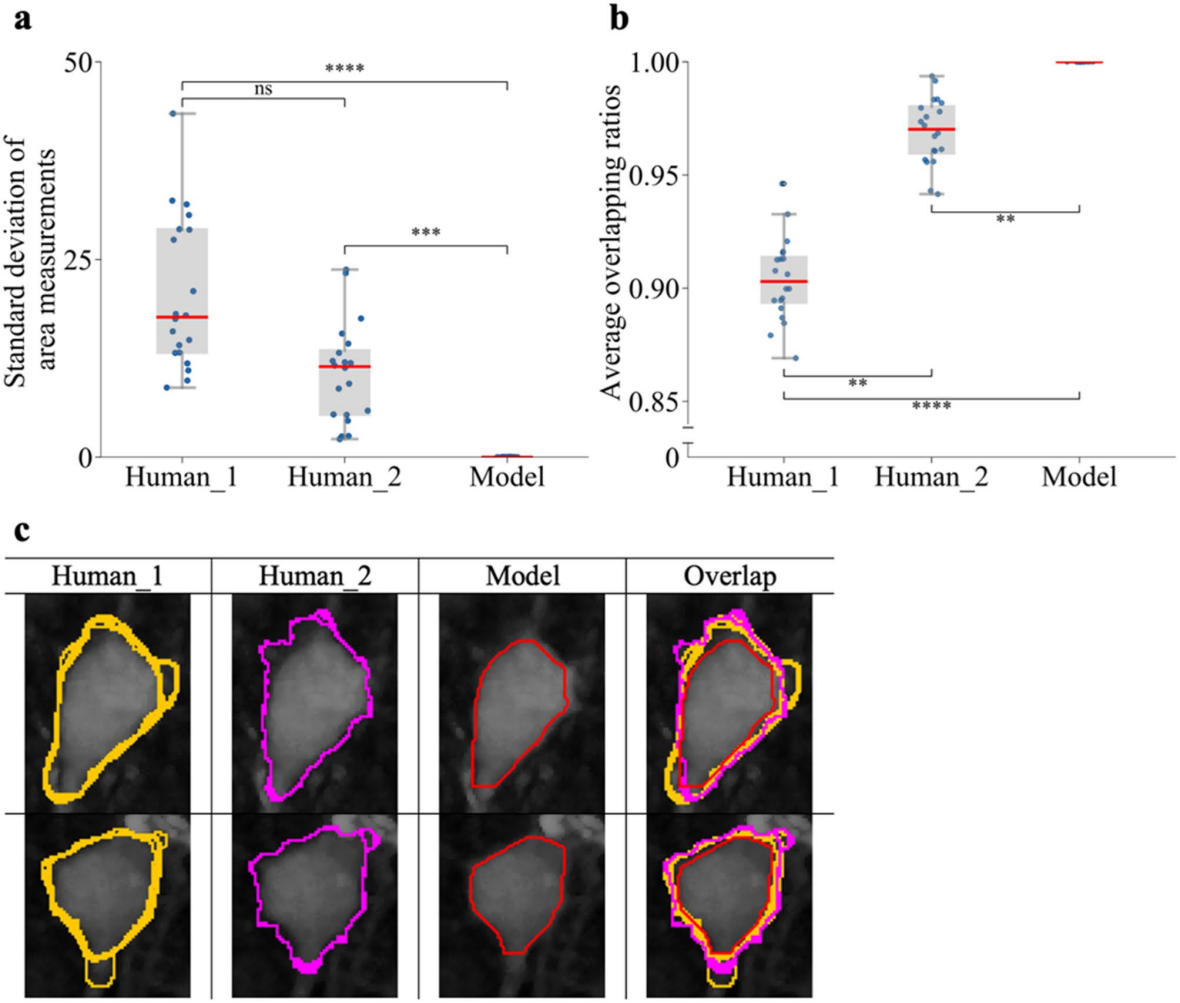
Somatic segmentation by the ANMAF framework lacked variability compared to human labelers. (**a**) The standard deviation of the somatic area measurements of each neuron obtained from two human annotators and the model (*p* < 0.0001, Friedman test, significance between both Human_1 and Human_2 compared to the model, *p* < 0.0001 and = 0.004 respectively, Dunn’s post-hoc test, *n* = 20 neurons, five consecutive days). (**b**) Average overlapping ratios on contouring somatic regions (see “Methods” section) for the annotators and model (*p* < 0.0001, Friedman test, significant differences between all groups, Dunn’s post-hoc test n = 20 neurons, five consecutive days). Y-axis is zoomed. (**c**) Two example neurons with five overlapping contours from two annotators and ANMAF. Note the identical overlap by ANMAF.

We next compared the variability and reproducibility of a given ANMAF model with human annotators in repeated measurements of the same set of somatic regions. We randomly selected twenty different neurons. Two human annotators with prior experience in contouring somatic areas were tasked to label repeatedly the twenty neurons each day for five consecutive days. The annotators did not have access to their previous annotations, and the experimental images were accessed in a fixed order. Similarly, ANMAF detected and segmented the same 20 cells’ somatic regions in five independent trials. Our results show that the human annotators exhibited a significant amount of variability in area measurements compared to ANMAF, which showed none during repeated measurements (*p* < 0.0001, Friedman test, significance between both Human_1 and Human_2 compared to the model, *p* < 0.0001 and = 0.0004 respectively, Dunn’s post-hoc test, *n* = 20; Fig. 4a). ANMAF also showed the highest reproducibility in terms of the geometry of contours, measured by the overlap of the five repeated annotations (see “Methods” section). ANMAF produced the same outlines each time for the same neuron, which was significantly different from both human annotators (*p* < 0.0001, Friedman test, significant differences between all groups, Dunn’s post-hoc test; Fig. 4b,c). The lack of variance in ANMAF detection is critical for experimentalist. It shows that if given a same neuron, ANMAF will produce the same result without variance, compared to human annotators. Thus, ANMAF's consistency in repeated measurements of the same neuron allows increased rigor compared to human labelers.

### Generalizability

**Figure 5:**
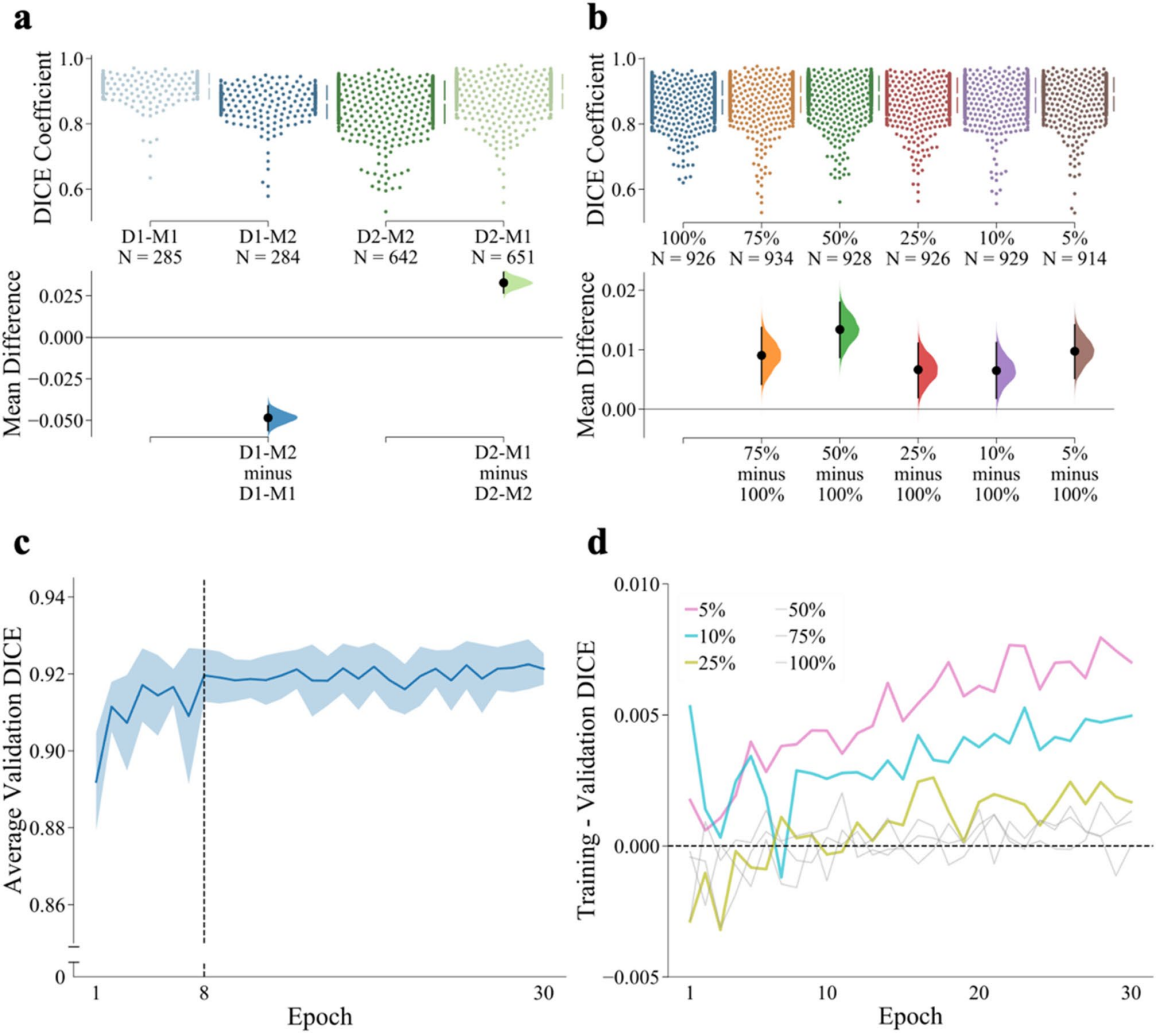
ANMAF was generalizable and trainable with a low number of human-labeled neurons in a few iterations. (**a**) Cumming estimation plot comparing the performance on learning synthetic data sets generated using different data sources. M1 was trained on synthetic images generated solely from somatic regions in D1, whereas M2 was trained on synthetic images produced using somatic areas from D2. D1 and D2 represent two different sets of Clomeleon expressing neurons in acute brain slices using two different two-photon microscopes. Upper axes, raw data. Each data point represents a matched somatic region between the human annotator and the model. SD are shown as gapped lines to each group’s right, and the mean of each group is indicated as a gap in the line. Each mean difference is plotted on the lower axes as a bootstrap sampling distribution. Mean differences of DICE coefficients between two groups are depicted as dots; 95% confidence intervals are indicated by the ends of the vertical error bars. N represents the number of human-annotated neurons that a model detects. (**b**) Cumming estimation plot comparing the performance on learning synthetic data sets generated using a different number of real neurons from D1 and D2. Graph details as A. (**c**) Average performance of the different ANMAF models (solid blue line). The shaded blue area covers one standard deviation below and above the average performance. (**d**) Difference between training and validation DICE coefficients when using a different number of training neurons. Note that the highest difference with 5% and 10% represents a thousandth of a unit change in the DICE coefficient.

Cell images produced by individual laboratories can differ in fluorescence intensity, background level, cell size, and resolution. Next, we tested how generalizable ANMAF was to various imaging devices, sequences, and protocols. We used two different data sets, namely, D1 and D2, containing Clomeleon (YFP channel) expressing neurons from acute brain slices acquired using two different microscopes and times (see “Methods” section). Using these data sets, two identical models, namely M1 and M2, were trained. We trained M1 on synthetic images generated solely from neuronal somatic regions in D1, whereas M2 was trained on synthetic images produced using somatic regions from D2. Model parameters, imaging preprocessing methods, and other settings were the same for both models. We then tested both models using real images in D1 and D2. M1 and M2 detected a similar number of manually annotated neurons in the real images of D1 and D2 (Table 1). While the differences in the average conformity of the contours were statistically significant in both D1 (mean difference − 0.048, CI [− 0.056, − 0.041]; *p* < 0.0001, Mann–Whitney test; Fig. 5a, Table 1) and D2 (0.033, CI [0.027, 0.039]; *p* < 0.0001, Mann–Whitney test; Fig. 5a, Table 1), they were not distinctive in practice. For example, neuronal regions in D1 were 237 μm^2^ on average, and a DICE coefficient difference of 0.02 corresponded to just a 6 μm^2^ variation in the area. Overall, M1 and M2 had a similar performance in D1 and D2. These results demonstrated that the training of ANMAF with one imaging protocol can be generalizable to an unseen data set from a different image protocol.

**Table 1:** Percentage of matched cells and average DICE coefficient of two ANMAF models.

Data	M1	M2	Human detected neurons	M1 detected neurons	M2 detected neurons
D1	100% (0.9151)	99.65% (0.8666)	285	1951	1973
D2	95.74% (0.8989)	94.41% (0.8661)	680	1885	1881

M1 and M2 columns present the percentage of manually annotated somatic regions in the datasets D1 and D2 that ANMAF detected, respectively. Parentheses represent the average DICE coefficients between manual and model-generated contours of matched somatic areas. The last three columns demonstrate the total number of neurons labeled by human annotator, M1, and M2, respectively.

### Minimum supervision

Finally, we evaluated the minimal number of human-labeled neurons that are necessary for training ANMAF. Six identical models were trained on six different synthetic data sets that were generated by using 653 (100%), 490 (75%), 327 (50%), 164 (25%), 66 (10%), and 33 (5%) human-annotated neurons, respectively. Training parameters and preprocessing methods remained the same for the six models. All models detected a similar number of manually annotated cells (Table 2). There were statistically significant differences in the average conformity of contours for models using 75%, 50%, 25%, 10%, and 5% compared with that of 100% (mean difference from 0.007 to 0.013, CIs [0.002, 0.018], p < 0.0001 for all five comparisons, Mann–Whitney test; Fig. 5b, Table 2). However, the corresponding differences in area measurements were not discernible. During training, these six models’ performance did not improve by increasing the number of training epochs (Fig. 5c). Instead, there was a tendency towards overfitting for models using less than 25% labeled neurons (Fig. 5d). These results indicate that the ANMAF can learn to detect and contour somatic regions even with a small number (as low as 33) of human-labeled neurons and does not necessitate many training epochs.

**Table 2:** Percentage of matched cells and average DICE coefficient of ANMAF models trained on synthetic images generated using a different number of neurons in datasets D1 and D2.

Data	100%	75%	50%	25%	10%	5%
# of cells	653	490	327	164	66	33
D1	99.65% (0.8818)	100% (0.8968)	100% (0.9008)	100% (0.8924)	100% (0.8912)	100% (0.8940)
D2	94.41% (0.8808)	95.15% (0.8872)	94.41% (0.8916)	93.97% (0.8856)	94.56% (0.8860)	92.21% (0.8894)

Parentheses represent the average DICE coefficients between manual and model-generated contours of matched somatic regions.

## Discussion

Here, we developed a novel high-throughput Mask R-CNN-based framework, named ANMAF, to automatically contour neuronal somatic areas of fluorescent protein-expressing neurons in acute brain slices. ANMAF detected neuronal somatic regions with a performance comparable to human annotators with high precision and consistency. Also, ANMAF trained with a data set from one imaging protocol was generalizable to an unseen data set from a different protocol and was trainable with as low as 33 (5%) human-labeled neurons.

The main contribution of this study is as follows. First, we proposed a novel and generic framework, enabling the application of CNNs to neuronal morphology analysis even under situations where there is an insufficient number of training samples or there is incomplete human annotation for every neuron in each image. Second, we conducted a quantitative study to determine ANMAF’s efficacy, reliability, and generalizability. We found that ANMAF accurately detects a larger number of neurons than humans in a fraction of time, has high reliability showing a minimal variance when presented with the same neuron, and is generalizable to different imaging protocols.

Prior automated techniques to detect somatic bodies have used thin fixed tissue slices with tagged or injected fluorophores and confocal microscopy^7,22,23^ or transmitted electron microscopes^12^. Other developed algorithms have been evaluated on neuronal cultures or thin brain tissue using few neurons^8,9^. However, thick brain slices (350 microns or more) commonly used in physiological studies have significant challenges, including overlaps among neurons across different depth planes, changes in fluorescence intensity throughout the thickness of a brain slice, low contrast between cell bodies, and the background, among others. While CNNs have been used to detect neurons from thick brain slices, they have required extensive training sets^24^, semi-supervised regularization^10^, or a hybrid architecture based on Morse theory and deep net architectures^11^. Our ANMAF has the advantage of being trainable with a minimal number of human-labeled neurons, using a simplified architecture, and producing reliable area measurements when using thick acute brain slices.

Our proposed framework has three small limitations to be addressed in the future. First, we observed that ANMAF had a slight tendency to underestimate darker neurons’ somatic area and overestimate brighter neurons than manual measurements. Introducing an image normalization technique to the preprocessing stage could help mitigate this issue. Second, our current framework lacks the encoding of rotation and flip invariance into CNN. Adding mechanisms for handling these transformations can help produce more robust contours of somatic regions against perturbations. Third, when contouring a brain Z-section, human annotators were able to examine its adjacent slices. However, the current framework does not take the contextual information across slices into consideration. Also, the neuronal volume may be a more accurate estimate of somatic size compared to the area. Therefore, modifying the current framework to address these limitations is the next step.

In conclusion, we present a valid, reliable, and generalizable Mask R-CNN framework (ANMAF) to automatically contour fluorescent neuronal somatic areas in acute brain slices. Our framework will significantly contribute to the neuroscience field by detecting and labeling many neurons in an unbiased way leading to a more rigorous and quantitative study of neuronal morphology. The model will permit quantitative measurement of the changes in neuronal area during different physiological and pathological insults using many neurons without the cost of manual annotation, which may lead to selection bias, inter and intra-labeler variance, and limited and incomplete labeling. To facilitate future research, we implemented ANMAF to produce contours in an ImageJ compatible format and release the source code free for research purposes (https://github.com/stephenbaek/ANMAF).

## Methods

### Animals and brain slice preparation

Postnatal CLM-1 Clomeleon mice (P7–P60; C57bl/6 background, a generous gift of Kevin J. Staley and also obtained from JaxLab B6.Cg-Tg(Thy1-Clomeleon)1Gjau/J^15^) of both sexes were anesthetized using inhaled isoflurane and decapitated per protocol, approved by the Institutional Animal Care and Use Committee of the University of Iowa and Massachusetts General Hospital Center for Comparative Medicine. All methods and experiments were performed in accordance with the relevant guidelines and regulations including ARRIVE (https://arriveguidelines.org). We used 4 mice for D1 dataset and 3 mice for D2 dataset. An additional mouse was used for variability comparison between human and model. The brain was removed and placed in ice-cold artificial cerebrospinal fluid (aCSF) containing (in mM) NaCl (120), KCl (3.3), CaCl_2_ (1.3), MgCl_2_ (2), NaH_2_PO_4_ (1.25), NaHCO_3_ (25), and D-glucose (10) with pH 7.3–7.4 when bubbled with 95% O_2_ and 5% CO_2_. Coronal brain slices, 350–450 μm thick, were cut using a vibratome (Leica VT1000S) while submerged in aCSF containing 2 mM kynurenic acid. The brain slices were placed in an interface holding chamber containing aCSF at room temperature for 30 min. Next, the temperature was slowly increased and held at 30 °C after that. Slices were stored for one hour minimum before being transferred to the recording chamber.

### Imaging

We imaged neurons on two different two-photon microscopes: (A) a Fluoview 1000MPE two-photon microscope with pre-chirp optics and a fast acoustic-optical modulator mounted on an Olympus BX61WI upright microscope body with a 25× water immersion objective (NA 1.05) (dataset D1) with two photomultiplier tubes (Hamamatsu Photonics). (B) a Bruker Ultima In Vitro galvo-resonant system mounted on an Olympus BX51WIF upright microscope body with a 20× water immersion objective (NA 1.00) with two Hamamatsu Photonics GaAsP end-on photomultiplier tubes (dataset D2). A Ti: sapphire mode-locked laser (DeepSee Mai Tai for the Fluowiew or a Mai Tai HP DS for the Bruker Ultima; Spectra-Physics) generated two-photon fluorescence with 860 nm excitation. Emitted light was bandpass filtered through two emission filters: 460–500 nm for cyan fluorescence protein (CFP) and 520–560 nm for yellow fluorescence protein (YFP). For our experiments, we exclusively used the YFP channel as we are interested in somatic area measurements. Slices were perfused with aCSF, held at 32–34 °C, and aerated with 95% O_2_–5% CO_2_. Three-dimensional stacks (3D) of raster scans in the XY plane with a resolution of 512 × 512 pixels were imaged at a z-axis interval of 2 μm.

### Manual detection and measurements of neuronal cell bodies

Measurements of the neuronal area of 3D stacks were performed using ImageJ (National Institutes of Health) as described previously^4,16^. Briefly, we loaded YFP z-stack images and subtracted the corresponding background level from the entire 3D stack. Next, 3D planes were median filtered. Maximum intensity Z-projections were created every 10 μm for neuronal area determination. This projection allowed us to have, in a single plane, the maximal area projection for each neuron. A region of interest (ROI) was drawn manually around the cell bodies based on an automatic neuronal edge detection plugin in ImageJ (‘Canny Edge Detector’ plugin).

### Automatic detection and measurements of neuronal cell bodies

#### Synthetic images generation and preprocessing

We constructed a library of background tiles by randomly sampling empty (background-only) regions from the original microscopy images. A total of 50 tiles were collected in this study. The sampled background tiles were then randomly sampled and placed in a grid to form a synthetic background image. We placed the tiles with small overlaps and blended them via linear pixel value interpolation for a smooth, seamless transition between tiles. A total of 500 synthetic backgrounds were generated as such (512 $\times$ 512 pixels). These artificial backgrounds were augmented by randomly changing the average brightness of the pixel values and by adding Gaussian random noise to increase diversity. Onto such synthetic backgrounds, somatic images were then digitally transplanted by copying image pixel values within somatic regions from real microscopic images to synthetic background images. Although a somatic region could be pasted at arbitrary locations of randomly selected artificial backgrounds, the algorithm avoided placing it at locations that already contained other areas. During the transplantation, one pixel of padding was applied from the manually annotated cell boundaries so that the padded regions could be blended into the background, similarly to how tiles were blended. The periphery of the cells was smoothly blended into the background by varying the transparency (image alpha), in which the transparency was inversely proportional to the distance from the cell boundaries: pixels near the cell boundary were opaque while the ones away from the edge were entirely transparent. This was to implement a more natural-looking boundary between the transplanted cells and synthetic backgrounds. During the transplantation, the diversity in training images was achieved by transforming the cell regions being transplanted, with random rotations of 90 degrees, left–right flips, bottom-up flips, arbitrary resizing between 0.8 and 1.2 scale factors, and random cell brightness between 0.95 and 1.3.

#### Mask R-CNN framework

**Figure 6:**
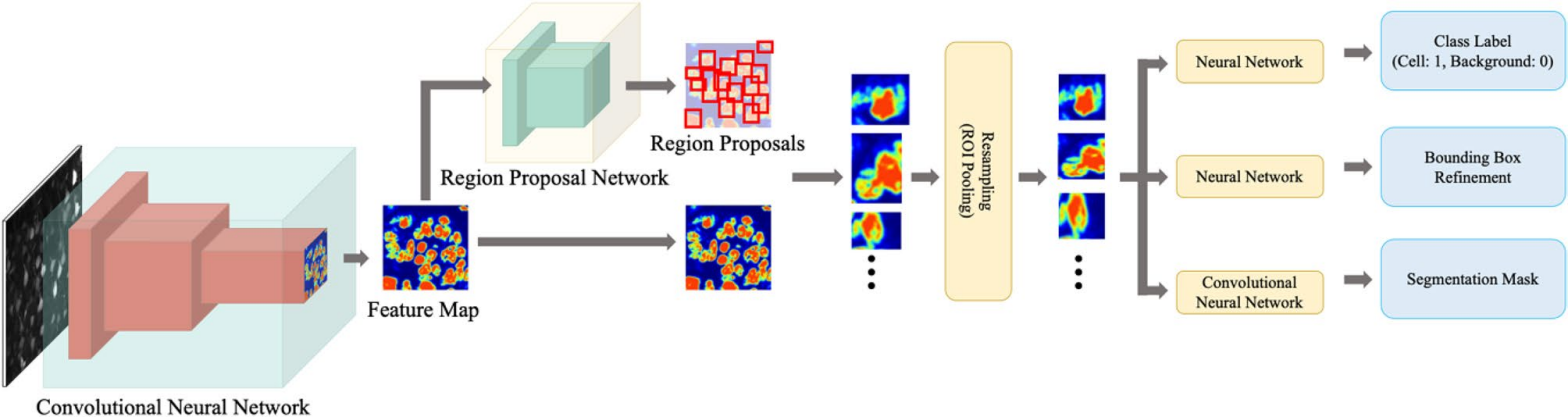
Mask R-CNN architecture. The architecture took a cell image as an input, which was then processed by a convolutional neural network to generate feature maps. Each pixel of the feature maps represented an artificial neuron’s activation covering a localized receptive field at the corresponding spatial location. The feature maps were then passed to the region proposal network (RPN), which processed candidate regions (red boxes) where soma might exist. This made the search process more efficient compared to an exhaustive search. The region proposals were then resampled to square-shaped image patches to make them compatible with the later processes. After resampling, three different artificial neural networks were applied to square patches: one for determining whether the region proposal contains a soma or not; another for refining the bounding boxes to fit the soma more tightly; and the other for generating segmentation masks of somas.

Before training the Mask R-CNN model, images were preprocessed by applying the contrast limited adaptive histogram equalization^25^ algorithm to normalize the cell brightness variations across different image sub-regions. The original grayscale images were resized to 1024 × 1024 pixels and duplicated into three red–green–blue (RGB) channels to make them compatible with the Mask R-CNN model pre-trained on a generic computer vision object segmentation data set, called Common Objects in Context (COCO) data set^17^. In the Mask R-CNN implementation, we used the residual network^26^ with 101 layers (ResNet-101) as a backbone, which generated a rich set of preliminary feature maps from a microscopic image. The feature maps generated by RestNet-101 contained numerical encodings of various geometric characteristics at different locations of the input cell image. These pieces of information were then processed in the second step by another block of the CNN module, called the region proposal network (RPN), in which bounding boxes around candidate soma instances were detected. The RPN made many highly sensitive yet less specific (many false positives) proposals on each region in the cell image where soma may be present. These region proposals, which varied in shape, were then warped and resampled to fixed-size square-shaped image patches in the third step. At the last stage, these resampled patches were fed into three additional branches of CNNs: the first branch of CNN determined whether the region proposal contained a soma or not to eliminate false-positives; the second branch of CNN refined the bounding boxes proposed by the RPN to fit the soma region tighter and more precise; lastly, the third branch generated a pixel-wise binary segmentation mask indicating which pixel contains an image of the soma (Fig. 6).

#### Training of mask R-CNN

During training, a multi-task loss was defined on each resampled patch as:

$$L= \alpha {L}_{class}+\beta {L}_{bounding\_box}+\gamma {L}_{mask}.$$

The first task loss ${L}_{class}$ is the binary cross-entropy loss for classification on whether a region proposal contains a soma or not. The second task loss ${L}_{bounding\_box}$ is the smooth-L1 loss for measuring how well a bounding box proposed by the RPN fits the actual soma region. The third task ${L}_{ mask}$ is the average binary cross-entropy loss over each pixel in a pixel-wise binary segmentation mask of a proposed soma region. $\alpha$, $\beta ,$ and $\gamma$ are weights on these three tasks loss.

Our mask R-CNN models were trained through backpropagation using a stochastic gradient descent optimizer with a 0.001 learning rate, 0.9 learning momentum, and 0.0001 weight decay. We used a mini-batch size of 4 with 500 training steps per epoch for 30 epochs. Three thousand synthetic images with different configurations were generated for tuning each model’s weights, and a validation set of 1000 synthetic images was used for monitoring the training progress. We ensured that we used disjoint sets of somatic areas to produce synthetic data sets for training and validation. Each model was trained on a machine equipped with 56 cores, 512 GB memory, and one Tesla P100-PCIE-16 GB GPU for around 6 h.

#### Evaluation metrics

The DICE coefficient between two segmentation masks (created based on contours) was defined as:

$$DICE\left(X,Y\right)=\frac{2|\text{X}\cap \text{Y}|}{(|\text{X}|+|\text{Y}| )},$$

where $|X|$ and $|Y|$ are the number of pixels belonging to each mask. The DICE coefficient equals to twice the number of pixels common to both masks divided by the sum of the number of pixels belonging to each mask. A value of 1 indicates identical segmentations, while 0 means no pixels in common.

The shoelace algorithm, applied on the contour of a somatic region to compute its area, was defined as:

$$Area= \frac{1}{2} \left|\sum_{i=1}^{n-1}{x}_{i}{y}_{i+1}+ {x}_{n}{y}_{1}- \sum_{i=1}^{n-1}{x}_{i+1}{y}_{i}- {x}_{1}{y}_{n}\right|,$$

where $\left({x}_{i}, {y}_{i}\right), i=\text{1,2},\ldots , n$ are the ordered vertices of the polygon (contour).

As there were multiple measurements for every neuron from each annotator in repeated measurements, we quantified the reproducibility of a human annotator and ANMAF in any one neuron using the neuron’s average overlapping ratio, which was defined as:

$$AvgOverlapping \left({X}_{1}, \cdots {X}_{5}\right)= \frac{1}{5}{\sum }_{i=1}^{5}\frac{\left|({\bigcap }_{i=1}^{5}{X}_{i})\right|}{\left|{X}_{i}\right|} ,$$

where $\left|({\bigcap }_{i=1}^{5}{X}_{i})\right|$ is the number of pixels common to five segmentation masks (created based on five contours) of one somatic region and $\left|{X}_{i}\right|$ is the number of pixels belonging to $ith$ segmentation mask.

### Statistics

We tested the Gaussian distribution of data with the Shapiro–Wilk and Kolmogorov–Smirnov tests. Paired *t*-tests were used for parametric comparisons, and the Mann–Whitney test was used for unpaired non-parametric data. For non-parametric analysis, a Friedman test was performed with Dunn’s multiple comparison test for post-hoc analysis. Estimation statistics were performed and used to compute 95% confidence intervals of the mean differences^27,28^. Statistical significance was set to *p* < 0.05. IgorPro v8.04 (WaveMetrics), Prism 8 (GraphPad Software, LLC), and the python package DABEST^28^ were used for data analysis.

## References

[1] Andrew RD, Labron MW, Boehnke SE, Carnduff L, Kirov SA (2006). Physiological evidence that pyramidal neurons lack functional water channels. Cereb. Cortex.

[2] Risher WC, Andrew RD, Kirov SA (2009). Real-time passive volume responses of astrocytes to acute osmotic and ischemic stress in cortical slices and in vivo revealed by two-photon microscopy. Glia.

[3] Glykys J (2017). Chloride dysregulation, seizures, and cerebral edema: A relationship with therapeutic potential. Trends Neurosci..

[4] Glykys J, Duquette E, Rahmati N, Duquette K, Staley KJ (2019). Mannitol decreases neocortical epileptiform activity during early brain development via cotransport of chloride and water. Neurobiol. Dis..

[5] Rungta RL (2015). The cellular mechanisms of neuronal swelling underlying cytotoxic edema. Cell.

[6] Murphy TR (2017). Hippocampal and cortical pyramidal neurons swell in parallel with astrocytes during acute hypoosmolar stress. Front. Cell. Neurosci..

[7] Falk T (2019). U-Net: Deep learning for cell counting, detection, and morphometry. Nat. Methods.

[8] Ozcan B, Negi P, Laezza F, Papadakis M, Labate D (2015). Automated detection of soma location and morphology in neuronal network cultures. PLoS ONE.

[9] Kayasandik CB, Labate D (2016). Improved detection of soma location and morphology in fluorescence microscopy images of neurons. J. Neurosci. Methods.

[10] Xu, K., Su, H., Zhu, J., Guan, J.-S. & Zhang, B. Neuron segmentation based on CNN with semi-supervised regularization. In *2016 IEEE Conference on Computer Vision and Pattern Recognition Workshops (CVPRW)* 1324–1332 (IEEE, 2016). 10.1109/CVPRW.2016.167.

[11] Banerjee S (2020). Semantic segmentation of microscopic neuroanatomical data by combining topological priors with encoder-decoder deep networks. BioRxiv..

[12] Hosseini SMH, Chen H, Jablonski MM, Gimi BS, Krol A (2020). Automatic detection and counting of retina cell nuclei using deep learning. Medical Imaging 2020: Biomedical Applications in Molecular, Structural, and Functional Imaging.

[13] He, K., Gkioxari, G., Dollar, P. & Girshick, R. Mask R-CNN. In *2017 IEEE International Conference on Computer Vision (ICCV),* Vol. 2017, 2980–2988 (IEEE, 2017).

[14] Durkee MS, Tomaszewski JE, Ward AD (2020). Improved instance segmentation of immune cells in human lupus nephritis biopsies with Mask R-CNN. Medical Imaging 2020: Digital Pathology.

[15] Kuner T, Augustine GJ (2000). A genetically encoded ratiometric indicator for chloride. Neuron.

[16] Glykys J (2009). Differences in cortical versus subcortical GABAergic signaling: A candidate mechanism of electroclinical uncoupling of neonatal seizures. Neuron.

[17] Lin, T.-Y. *et al.* Microsoft COCO: Common objects in context. In *Lecture Notes in Computer Science (Including Subseries Lecture Notes in Artificial Intelligence and Lecture Notes in Bioinformatics)* Vol. 8693, LNCS 740–755 (2014).

[18] Polyak BT (1964). Some methods of speeding up the convergence of iteration methods. USSR Comput. Math. Math. Phys..

[19] Dice LR (1945). Measures of the amount of ecologic association between species. Ecology.

[20] Braden B (1986). The surveyor’s area formula. Coll. Math. J..

[21] Warfield SK, Zou KH, Wells WM (2004). Simultaneous truth and performance level estimation (STAPLE): An algorithm for the validation of image segmentation. IEEE Trans. Med. Imaging..

[22] Luengo-Sanchez S (2015). A univocal definition of the neuronal soma morphology using Gaussian mixture models. Front. Neuroanat..

[23] Cameron W, Bui C, Bennett A, Chang H, Rocheleau J (2020). Cell segmentation using deep learning: Comparing label and label-free approaches using hyper-labeled image stacks. BioRxiv..

[24] Soltanian-Zadeh S, Sahingur K, Blau S, Gong Y, Farsiu S (2019). Fast and robust active neuron segmentation in two-photon calcium imaging using spatiotemporal deep learning. Proc. Natl. Acad. Sci..

[25] Zuiderveld K, Zuiderveld K (1994). Contrast limited adaptive histogram equalization. Graphics Gems.

[26] He, K., Zhang, X., Ren, S. & Sun, J. Deep residual learning for image recognition. In *2016 IEEE Conference on Computer Vision and Pattern Recognition (CVPR)* 770–778 (IEEE, 2016). 10.1109/CVPR.2016.90.

[27] Calin-Jageman RJ, Cumming G (2019). The new statistics for better science: Ask how much, how uncertain, and what else is known. Am. Stat..

[28] Ho J, Tumkaya T, Aryal S, Choi H, Claridge-Chang A (2019). Moving beyond P values: Data analysis with estimation graphics. Nat. Methods.

